# Loneliness and Social Isolation of Older Adults and Quality of Dyadic Relationships with Migrant Domestic Workers: A Mixed-Method Study †

**DOI:** 10.3390/healthcare12242496

**Published:** 2024-12-10

**Authors:** Maria Shuk Yu Hung, Ken Hok Man Ho, Michael Man Ho Li, Edward Kwok Yiu Choi

**Affiliations:** 1 S.K. Yee School of Health Sciences, Saint Francis University, Hong Kong, China; 2 School of Nursing and Midwifery, La Trobe University, Melbourne, VIC 3086, Australia; k.ho@latrobe.edu.au

**Keywords:** loneliness, older adults, dyadic relationship quality, foreign domestic helpers, migrant domestic workers

## Abstract

Background/Objectives: Loneliness among older individuals is widespread globally, leading to increasing public health and policy concerns. Migrant domestic workers (MDWs) offer continuous services for older adults worldwide, recompensing for dwindling support from family members. The study objectives were to explain how the quality of dyadic relationships with MDWs is associated with older adults’ loneliness and further explore older adults’ perceived experiences of care by MDWs. **Methods:** A mixed-methods study was conducted from 2021 to 2023. Convenience and snowball samplings of older adults aged ≥60 living with MDWs were recruited by elderly community centers in Hong Kong for a survey, and purposive sampling of in-depth interviews followed. The survey comprised several well-validated scales assessing loneliness, perceived social networks, and the quality of dyadic relationships. Face-to-face in-depth interviews were audio-recorded and transcribed for preliminary content analysis. A total of 288 older adults participated in the first phase of the survey interviews. Among them, 19 joined the in-depth interviews in the second phase. **Results:** There is a high prevalence of loneliness among older adults being cared for by MDWs in Hong Kong. In addition, the results showed that social loneliness negatively and significantly correlated with perceived dyadic relationship quality and social network. Two major interrelated themes were identified along with eight subthemes: (1) established relational interaction with MDWs and (2) enjoyed functional assistance and support from MDWs. **Conclusions:** Our study findings illustrated that good dyadic relationships among MDWs and older adults enhance older adults’ social connections and networking, alleviating loneliness and social isolation. Appropriate strategies are suggested to strengthen older adults’ social support and improve the quality of dyadic relationships with MDWs, which may reduce loneliness among older adults.

## 1. Introduction

Social isolation and loneliness among older individuals are widespread globally and cause serious health consequences for their mental and physical health, well-being, and quality of life [1], which has led to increasing public health and policy concerns, especially in light of the COVID-19 pandemic in the past few years [1,2,3,4,5]. Social isolation suggests infrequent social contact or networking with others, and loneliness implies the subjective feeling of being lonely or isolated [6]. During the pandemic, the inevitable city lockdown and execution of social distancing policies further aggregated the serious public health risks and consequences among considerably older individuals worldwide [2,5]. Studies found that older adults’ loneliness risk factors are frequently associated with being single/widowed, having a deficient social network, lack of social activity, self-perceived unhealthiness, and feeling depressed/depressive mood [7].

According to WHO (2021), up to one in three older people are lonely in various countries worldwide, e.g., in China, Europe, Latin America, and the United States of America. However, family support significantly protects against loneliness in older Chinese adults in tradition [8]. Local studies found that about 30–40% of older Chinese adults in Hong Kong experienced moderate to severe loneliness [9], and insufficient family and community support has intensified loneliness in recent years [10]. Nowadays, various factors, such as increased life expectancy, limited living space, low birth rates, and a shortage of family caregivers, may decrease family members’ capacity or availability to support the well-being of older adults [11].

Migrant domestic workers (MDWs) are crucial to offering continuous services and social interaction for older adults worldwide, recompensing for dwindling support from family members [11,12,13,14,15,16,17]. Moreover, due to the increasing trend of shifting to home-based elder care in the family, there is a remarkable need for MDWs in Asian countries, including Hong Kong [11,16,17,18]. By the end of 2023, about 356,000 MDWs were working in Hong Kong, 56% and 42% of whom were from the Philippines and Indonesia, respectively [19]. The primary reasons for the employment of MDWs were the foreseeable benefits for older adults and insufficient institutional service [13]. The MDWs’ responsibilities are not stipulated but are based on the individual needs of the employers [20]. Evidence shows that live-in domestic helpers could provide functional support such as daily housework, person-centered care, and escort services for older adults [18,21]. However, some researchers have disputed that engaging MDWs in a family may predispose feelings of loneliness among older adults [22].

According to the convoy model of social relations, the people surrounding an individual form a dynamic network, which influences an individual’s wellbeing [23]. Our preliminary findings on 178 older adults showed that poor relationships between older adults and MDWs are associated with loneliness and social loneliness, but not the emotional loneliness of older adults [12]. Social loneliness refers to the absence of valued social networks, while emotional loneliness refers to the absence of an intimate figure [24]. However, another study showed that living with MDWs is negatively associated with the emotional loneliness of older adults [9]. As such, it is assumed that the relationship between older adults and MDWs will influence the level of loneliness of older adults. Furthermore, our qualitative studies showed that MDWs who committed to a relationship with older adults would help older adults maintain connections with others [11,25]. Therefore, it is assumed that the relationship between MDWs and older adults will influence the social networks of older adults, which are associated with loneliness [26]. Through a mixed-methods design, this study further aimed to explain how the quality of dyadic relationships with MDWs is associated with older adults’ loneliness and further explore older adults’ perceived experiences of care by MDWs. The quantitative survey of the study aimed to investigate the following hypotheses:

**H1:**  *Perceived dyadic relationship quality positively predicts overall loneliness/social loneliness/emotional loneliness.*

**H2:**  *The perceived social network plays an intermediary role in the relationship between the perceived dyadic relationship quality and overall loneliness, social loneliness, and emotional loneliness.*

**H3:**  *Perceived dyadic relationship quality positively predicts perceived social networks.*

Then, qualitative in-depth interviews were used to understand the dyad relationship that older adults experienced in the second phase. After a comprehensive understanding of how dyadic relationship quality influences older adults’ experiences of loneliness, strategies can then be developed to inform interventions to enhance positive relationship quality and reduce loneliness in older adult populations in the near future. The study results can also inform policymakers and community organizations to offer support and strategies to address loneliness among older adults if appropriate.

## 2. Materials and Methods

### 2.1. Design

We used an explanatory sequential mixed-methods design, with cross-sectional questionnaire interviews followed by in-depth qualitative interviews. This approach allowed the research team to comprehensively understand the collected data and the participants’ perspectives [27].

### 2.2. Subjects and Sampling

The potential participants were older adults aged 60 or above who could understand Chinese and who were living with and cared for by MDWs for daily living. They were recruited from elderly community centers across different districts of Hong Kong. As the social distancing policies were implemented during the COVID-19 pandemic in Hong Kong from 2021 to 2023, recruiting older adults for data collection was quite challenging, as the elderly community centers’ services were inevitably affected. Convenience and snowball sampling were employed. If the older adults were interested in joining, they were referred by center staff to the team.

In a study of 1144 older Chinese adults [28], an effect size of 0.0375 was found for the impact of subjective social relationships on older adult loneliness while controlling for five other predictors in the model. According to G-Power 3.1.9.4, at least 283 subjects should be recruited for a hierarchical multiple regression to assess the effect of perceived social relationship quality while controlling for ten predictors of the level of loneliness among older adults, with a 5% type I error (alpha) and a power of 90% [28].

### 2.3. Ethical Considerations

Ethical approval was obtained before the implementation of the study. The researchers explained the purpose of the research and provided detailed information for potential participants’ consideration. After they understood that their anonymity was guaranteed and that they had the right to refuse to answer any questions during the interviews, written informed consent was obtained from individual participants. All the research information was kept confidential and would be destroyed after seven years.

### 2.4. Quantitative Survey Interviews and Outcome Measures

Due to the termination of the social restriction policy upon the end of the COVID-19 pandemic, we were able to recruit 100 more participants on top of the 178 older adults in 2023 [12]. This allowed us to further test H2 in the quantitative arm and further strengthened the explanatory power of this mixed-methods study, which was unable to be achieved in our preliminary findings [12]. The questionnaire used for the interview comprised socio-demographics and several well-validated scales of the Chinese version, including the Lawton Instrumental Activities of Daily Living Scale, the De Jong Gierveld Loneliness Scale, the Mutuality Scale, and the Lubben Social Network Scale. The sociodemographic variables of age, gender, marital status, educational level, living arrangements, health status, MDWs’ nationality, spoken language, age, and serving years were asked. The 9-item Lawton Instrumental Activities of Daily Living Scale was used to evaluate the functional status of older adults [29]. The 6-item De Jong Gierveld Loneliness scale was used to assess the emotional and social loneliness perceived by older adults [30]. The 15-item Mutuality Scale explored the relationship between caregivers and care receivers in the dimensions of love, shared pleasurable activities, shared values, and reciprocity [31]. The 10-item Lubben Social Network Scale measured older adults’ social network support from family, friends, and interdependent relations [32].

All the survey interviews were conducted face-to-face in quiet rooms of the elderly centers with which older adults were affiliated or near their homes. The center staff assisted the MDWs or family members who accompanied the older adult participants, arranging for them to wait in another area. Without the possibility of interruption, both the interviewee and interviewer could engage in a free, natural, and confidential conversation. If applicable, appropriate distancing and adequate disinfection measures were maintained based on the local government’s social distancing policy. The interviews ranged from ~30 to 45 min. Upon completing the survey interviews, participants were asked whether they were willing to be contacted for the second phase of an in-depth interview if appropriate.

### 2.5. Quantitative Data Analysis

Descriptive statistics were presented by *n* (%) for categorical variables. Continuous variables were presented by the mean (SD). Pearson’s product-moment correlation coefficient was used to determine the relation between the variables. To test the mediation analysis, the SPSS PROCESS macro, Model 4 (Version 4.2; Hayes, UK, 2022), and the bootstrapping method [33] were used for mediation analysis. The analysis was adjusted by age, marital status, educational level, living with family, and functional capacity of older adults because these were consistent correlates of loneliness [26], and the MDW’s nationality, spoken language, and serving years were also adjusted because they were associated with loneliness of older adults [12]. The bootstrapping with bias correction (BC) method was further used to compute the 95% confidence interval. Statistical analysis was performed using IBM SPSS Statistics for Windows, Version 26.0 (IBM Corp., Armonk, NY, USA computer software). A *p*-value of <0.05 was considered statistically significant.

### 2.6. Qualitative Interviews

Participants who experienced different levels of loneliness were invited to participate in the in-depth interviews using an interview guide with questions that included, for instance, “Can you share your daily life with your MDW?” and “Please tell me more about your experiences of how an MDW cares for your daily life”. Follow-up questions were asked as appropriate. The interviews were audio-recorded with participants’ consent. They focused on the dynamics of the participants’ relationships with MDWs and older adults experiencing loneliness. The participants were asked to share their experiences concerning their daily lives and relationships with MDWs. The researchers reviewed the first semi-structured interview transcript for interview skills, text accuracy, and overall understanding and made minor adjustments before the following interviews. Field notes were taken to document the participants’ demographics, emotions, gestures, physical expressions, and the researcher’s impressions during and after the interviews. The interviews were continued until data saturation.

### 2.7. Qualitative Data Analysis

After 19 interviews, the data were saturated. The interviews lasted from 25 to 70 min. All audio-recorded interviews were transcribed for data analysis. The field notes were incorporated into the verbatim transcripts. Using content analysis for the initial data analyzed, the interviews were read through a few times for a comprehensive meaning [34]. The first and third authors independently scrutinized the first four transcribed verbatim line by line separately to identify important ideas, including meaningful units. They then collaboratively labeled the texts into codes while retaining their essential meaning. A coding list was then developed to code the remaining verbatim transcriptions. Whenever there was information that did not match the coding list, the two authors collaboratively revised it. The participants’ narratives and ideas were further grouped into subthemes based on their similarities or differences. The two authors discussed the subthemes and came to a consensus. The revised subthemes with similar properties and dimensions were linked to form themes, providing a more comprehensive representation of the original concepts.

## 3. Results

### 3.1. Quantitative Results

In our study, 288 community-dwelling older adults from 34 community elderly centers participated. Table 1 shows the participants’ sociodemographic characteristics, health status, and co-living profile. Of the 288 participants, 247 (85.8%) were female, and 41 (14.2%) were male. The mean age was 82.77 (SD = 7.72); 106 (36.8%) were married, and 89 (30.9%) had attained secondary education or above. Additionally, 132 (45.8%) of the participants lived solely with MDWs, not their families. Most domestic workers were Indonesians (*n* = 227, 78.8%), and 234 (81.3%) spoke Cantonese.

Among 288 older adult participants, 128 (44.4%) who were cared for by MDWs had an overall loneliness score ≥ 2, which was considered lonely (≥2) on the 6-item DJGLS scale that ranged from 0–6 (three items for emotional and social subscales, respectively, ranged from 0–3), with a higher score indicating a greater level of loneliness. For the emotional and social subscales, scoring 1 or higher on these two subscales suggests being lonely emotionally or socially. A total of 131 (45.5%) had an emotional subscale score ≥ 1, and 129 (44.8%) had a social subscale score ≥ 1. In addition, 146 (50.7%) participants had an LSNS score < 20, which was considered at greater risk of social isolation. For the MS, which measures dyadic relationship quality, 93.8% (270 participants) scored less than 2.5, demonstrating poor mutuality among older adults and MDWs, as higher scores mean greater mutuality.

Correlations between perceived dyadic relationship quality or perceived social network and the outcome variables are presented in Table 2. Social loneliness had a negative and significant correlation with perceived dyadic relationship quality (r = −0.18, *p* < 0.01) and perceived social network (r = −0.20, *p* < 0.01). Perceived dyadic relationship quality had a positive and significant correlation with perceived social network (r = 0.31, *p* < 0.01).

**Figure 1:**
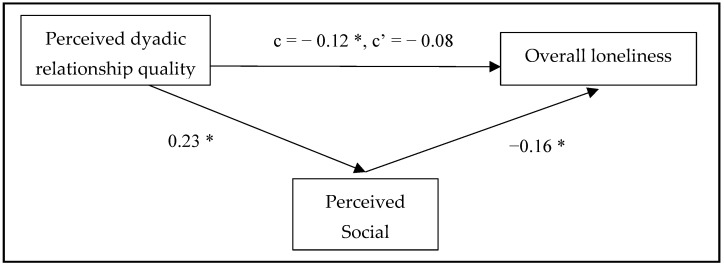
The mediation model for the relationship between perceived dyadic relationship quality and overall loneliness through perceived social network. Standardized coefficients of each arrow. c = total effect of perceived dyadic relationship quality on overall loneliness. c’ = direct effect of perceived dyadic relationship quality on overall loneliness. * *p* < 0.05.

**Figure 2:**
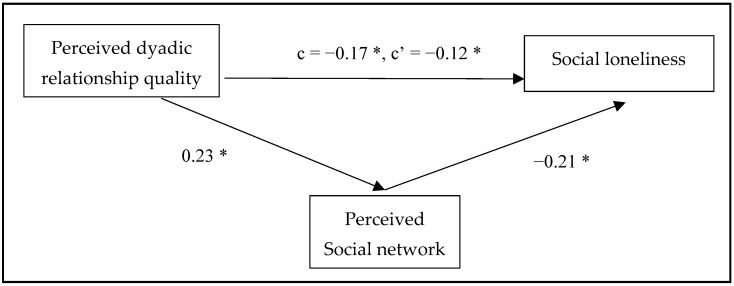
The mediation model for the relationship between perceived dyadic relationship quality and social loneliness through perceived social network. Standardized coefficients of each arrow. c = total effect of perceived dyadic relationship quality on social loneliness. c’ = direct effect of perceived dyadic relationship quality on social loneliness. * *p* < 0.05.

**Figure 3:**
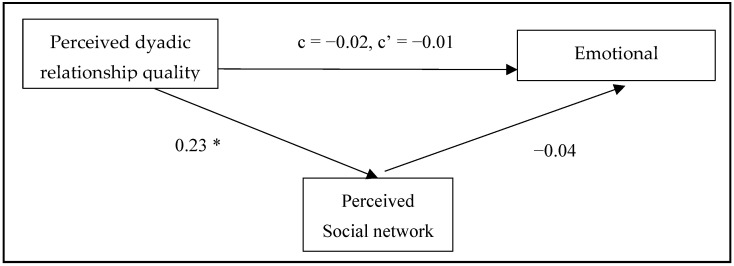
The mediation model for the relationship between perceived dyadic relationship quality and emotional loneliness through perceived social network. Standardized coefficients of each arrow. c = total effect of perceived dyadic relationship quality on emotional loneliness. c’ = direct effect of perceived dyadic relationship quality on emotional loneliness. * *p* < 0.05.

Mediation analysis was used to investigate H1–H3. The hypothesized research model indicated the perceived social network as a mediator in the relationship between perceived dyadic relationship quality and loneliness (overall/social/emotional), adjusted by age, gender, marital status, educational level, living arrangements, health status, and MDWs’ nationality, spoken language, age, and serving years. The results are summarized in Figure 1, Figure 2 and Figure 3.

Regarding H1, the results showed a significant total effect of perceived dyadic relationship quality on overall loneliness (*p* = 0.04) and social loneliness (*p* = 0.004). The direct effects in the models showed significant effects of perceived dyadic relationship quality on social loneliness (*p* = 0.044), but not on overall loneliness (*p* = 0.162). However, there is no significant total effect of perceived dyadic relationship quality on emotional loneliness (*p* = 0.720).

The results of H2 are shown in Table 3. The findings of mediation analysis indicated a significant indirect effect of perceived dyadic relationship quality on overall loneliness (indirect effect = −0.04, 95% CI: −0.07 to −0.01) and social loneliness (indirect effect = −0.05, 95% CI: −0.09 to −0.02) through the perceived social network. The results demonstrate statistical significance.

There is a significant association between perceived dyadic relationship quality and perceived social network, with β = 0.23, *p* < 0.001, confirming H3.

### 3.2. Qualitative Results

With reference to the above quantitative results collected and analyzed, we invited participants from the survey who had previously agreed to participate in the interviews, using purposive sampling with maximum variation. Of the 19 interview participants from eight different district/community elderly centers, 17 had various levels of loneliness (DJGLS score 1–6), 18 had poor mutuality with MDWs (MS < 2.5), and nine had great risk of social isolation (LSNS < 20). Five were male, one was single, nine were married, and nine were widowed. Five of them had secondary or above education. Twelve lived with the MDWs and family members, and seven lived with the MDWs only. The participants’ narratives were transcribed and analyzed. The qualitative findings illuminated the dyad relationship that older adults experienced through two major interrelated themes, (1) established relational interaction with MDWs and (2) enjoyed functional assistance and support from MDWs, with eight subthemes. The interrelated themes, subthemes, and examples of codes are shown in Table 4.

#### 3.2.1. Established Relational Interaction with MDWs

The established relational interaction illustrates the development of personal and social relationships among older adults and MDWs. Their relationships were dynamic and varied, occasionally becoming better or distant, influenced by different reasons or circumstances. The relation is related to the way or to the extent to which they directly interacted and communicated with each other. Although 18 out of 19 interviewees had poor mutuality with MDWs (MS < 2.5) in the survey stage before, we identified different relational interactions that might have exacerbated or alleviated loneliness depending on the quality of the relationship. Four subthemes of relational interaction include (1) approximate family companionship and care, (2) trust and sharing initiate emotional attachment, (3) merely employer-employee relations avoid interaction, and (4) behaviors influencing communication and relationships.

##### Approximate Family Companionship and Care

A few participants said they established a bond with the MDWs resembling a family relationship. They showed concern about each other’s health and well-being and demonstrated family companionship and care. A participant (P1) described her relationship with her MDW, who worked for her for four years. The MDW had delivered an unmarried baby a month ago.

*She worked for us (she and her husband) for four years and performed well. I treated her nearly as my daughter. She is unmarried but delivered a baby unnoticed last month. I felt shocked at that moment, but we still cared about her health and helped look after her newborn baby afterward. We shared her domestic work and taught her how to care for her baby. My husband likes the baby*. (P1)

The narrative reflected that acceptance and interdependence exist within relationships, with psychological, social, behavioral, and mutual support, especially in dealing with life challenges and hardship, better than in a formal employer-employee relationship. An aged couple (P11) appreciated her newly employed MDW, who was caring and considerate toward their health and well-being.

*At night, she purposely kept her room door open to quickly notice and be aware of whether we (the participant and her husband) needed help anytime. She used a fan rather than an air conditioner because she feared the temperature would be too cool and affect our health. She is also concerned about what food we prefer and what suits us.* (P11)

##### Trust and Sharing Initiate Emotional Attachment

Several participants believed that trust and sharing are essential in establishing long-term harmonious relationships. One participant (P14) emphasized that sharing, trusting, and appreciating the MDW’s performance could further enhance their relationship.

*She worked for years, and I felt grateful for her help during the pandemic. We occasionally chatted about my friends or her family. She is trustworthy and can handle everything, from housework to shopping for food. She worried for my safety when I went out. I occasionally apologized for my lousy temper towards her or forgot to give her money to buy food.* (P14)

When employers and MDWs establish trust in each other, they may provide emotional support during difficult times, representing personal care and concern. One lonely participant (P2) valued her MDW for listening and relieving her emotions (loneliness and unhappiness) in these few years.

*My husband does not care about or listen to me… My maid worked for three years, and we often chatted happily. When I was unhappy, I would speak, and she would listen, though she might not understand everything. She consoled me and supported my perspective, which made me feel a bit relieved from my loneliness and unhappiness.* (P2)

##### Merely Employer-Employee Relations Avoid Interaction

However, not all older adult participants have positive or harmonious relationships with their MDWs. Though they interacted respectfully, they maintained boundaries and did not share personal or family affairs. Participants admitted to having different roles and individual spaces for each other. One widower participant (P3), whose son is not living with him, stated the following:

*My son employed her (the MDW) to work for me. She only needs to fulfill her own housework responsibilities as a domestic worker. We seldom talk, and we will not disturb each other… Sometimes, her cooking does not suit my taste; I would not tell her. I usually go to the elderly centers and do not stay home during the day.* (P3)

The above case showed that the participant maintained distance from and avoided unnecessary communication with his MDW. Even though interactions were polite, they were limited to work-related matters. It was not uncommon in male participants in this study. Another participant (P8) also articulated similarly:

*I am approaching 90. My children employed her for us (and his wife). One day, we discussed her work practice and performance… I taught her how to do it, but she did not accept it. After that, I sometimes did housework myself, as her work did not fit what I wanted. Since then, I have kept our distance to avoid any conflict or argument with her.* (P8)

##### Behaviors Influence Communication and Relationships

Our participants emphasized mutual respect, loyalty, and honesty in fostering strong relationships with MDWs. They also noted that certain behaviors of the MDWs, such as dishonesty, impoliteness, and lack of communication, could significantly hinder or even damage their communications and relationships. Below are some specific instances shared by our participants:

*My previous domestic helper had broken the drawer and stolen my money before she left... We had reported to the police… The new helper conveyed an increase in her salary in the first few months after signing a 2-year contract, which made me feel unhappy and betrayed due to the breach of our consensus.* (P10)

*She (the MDW) is rude to me and talks back sometimes… She is dishonest. She pretended to go outside to buy stuff but met with her friends instead.* (P4)

*My maid is introverted, seldom talks, is unwilling to communicate, and uses her mobile phone constantly.* (P9)

#### 3.2.2. Enjoyed Functional Assistance and Support from MDWs

On the other hand, functional assistance and support by MDWs facilitated older adults with bodily deterioration or dysfunction and improved their personal and social lives, leading to decreased social loneliness. The four subthemes comprise (1) assisting in daily domestic work, (2) providing personal physical care and support, (3) accompanying on outings for activities for safety, and (4) (un)willing to work outside normal hours. Though 9 out of 19 interviewees reported a significant social isolation risk (LSNS < 20) in the survey stage, MDWs’ companionship might improve older adults’ social connections and loneliness through functional assistance and support for activities.

##### Assisting in Daily Domestic Work

Most participants appreciated their MDWs’ functional help, assistance with the housework, and relief of their workload, making them happy. One female married participant (P2), aged 84, verbalized the following:

*My daughter is not living with us, but she employed her (the MDW) to care for us (and her husband). She did most of the housework, cooking, and floor cleansing, assisted with our bathing… I am so happy that she relieved my burden as we age.* (P2)

Another participant (P10) living with family echoed the positive impact of the MDW on the family:

*The maid helped to cook the meals, perform the housework, and buy the stuff for the family. She offers great help with daily affairs. She also reminds us to have medications on time and measure blood pressure daily…* (P10)

However, a few participants were unsatisfied with the MDWs’ performance due to different standards, practices, or expectations. They felt that they should have shared or taken up a task, e.g., did not cook or cleanse well. One widowed participant (P9) living with her daughter complained, stating the following:

*Unlike previous domestic workers, she only cleanses the floors once a week, not daily. Although she cleansed the floor, it was still inadequately cleansed. I must do it again. I would rather not extend the maid’s contract after completion.* (P9)

##### Providing Personal Physical Care and Support

Personal physical care is significant for most participants in maintaining older adults’ physical health and delaying the deterioration of their physiological health. An 84-year-old widower (P16) without children employed an MDW by himself and expressed the following:

*I have chronic pain. I taught my maid how to perform massage for me to relieve pain, especially those areas I cannot massage himself, e.g., the neck and shoulder. She’s willing to help. As she learned the skills, she can perform massages in Indonesia after leaving Hong Kong. It is beneficial for her also.* (P16)

For those participants or couples with bodily dysfunction, the provision of personal care might facilitate support for mental health also. An aged couple (P11) treasured their maid, as seen below:

*I’m 85 with multiple chronic illnesses (hypertension, diabetes, heart disease…) and decreased physical ability … My husband has dementia and incontinence. I’m so worried…The maid was responsible and always willing to help change diapers for my husband at night. She helped me a lot.* (P11)

##### Accompanying Safety Outings for Activities

Poor physical or mental health might prevent participants from participating in outdoor activities. With the support and company of the MDWs, the perception of security and opportunities to participate in outdoor activities are increased, further improving social connections and increasing social support to alleviate participants’ loneliness and social isolation. A widow aged 83 described her health deterioration and fear as below:

*I’m a well-organized and scheduled person. I used to organize gatherings and outings with my friends… One day, I felt sudden dizziness, fainted outside, and was admitted to the hospital. It’s terrible. After that, I’m so scared to go outside alone. With her company (the MDW), I go here (the elderly center) or to the market, and she also reminds me to take my medication daily.* (P15)

A single participant (P13), whose sister-in-law employed an MDW for her, expressed her worries about safety and feelings of loneliness:

*I live in an old building, and most people leave. It’s so dark and quiet at night, and I’m frightened. I have poor vision…, and I cannot even see cars clearly when I cross the road. My sister-in-law employed a maid for me and accompanied me to the doctor and the elderly center…I feel safe… But she will leave next month, and I will be alone again...* (P13)

Another younger elderly (aged 67) living with her husband appreciated the MDW:

*I fell in 2020. In the following months after my operation, I frequently attended medical appointments with physiotherapy for rehabilitation in the hospital or clinics during the pandemic. I felt grateful for her companionship, as many domestic helpers were reluctant to go to the hospital, and some even left Hong Kong.* (P12)

##### (Un)Willing to Work Outside Normal Hours

Some participants, who were old or had physical problems living with their MDWs only, praised their helpers for being willing to help outside normal hours or during holidays (with payment). An old widow (age 87) (P18) who did not live with her children verbalized her nocturnal pain.

*I’ve chronic pain, and it hurts during sleep sometimes. I asked for her help to massage the muscle pain. She was willing to help even after sleeping at midnight. I could sleep again after the pain was reduced.* (P18)

However, another participant said her MDW was not committed to their agreed-upon work. A vulnerable participant (P15), who lives alone with chronic illness and functional problems and requires frequent admission or follow-up appointments in hospitals, expressed her dissatisfaction with her MDW as stated below:

*We mutually agreed that she (the MDW) would occasionally assist during the day off (with salary or compensation) if needed. I was admitted to the hospital for a few days and required her help for discharge home, but she did not help, which violated our agreement.* (P15)

In summary, our above quantitative results demonstrated that the quality of the dyadic relationship between MDWs and older adults would significantly influence their social networks, which were associated with loneliness. In addition, our qualitative studies, through analysis of participant narratives, further illustrated that MDWs who committed to a good relationship with older adults would facilitate and enhance older adults’ maintaining connections with society and others, alleviating loneliness and social isolation. This article is a revised and expanded version of a poster presentation at an international conference in Hong Kong [35].

## 4. Discussion

This is the first mixed-methods study about the dyadic relationship between older adults and MDWs and its influence on loneliness among older adults. In our study, the quantitative results demonstrate that among the 288 older adult participants, about half of them were found to be lonely (with DJGLS score: 44.4% overall score ≥ 2, 45.5% emotional score ≥ 1, 44.8% social score ≥ 1) and had social isolation with inadequate social engagement with their family and friends (50.7% had an LSNS score < 20). Our results reflect a higher level of loneliness and social isolation than the WHO (2021) declaration of 20–34% older persons in different countries worldwide and 30–40% locally [9]. This may be due to the influence of the social restrictions of the COVID-19 pandemic during the data collection. There are various factors related to loneliness, such as poor self-perceived health, functional and financial conditions, inadequate intimate relationships, poor relationship quality, ambivalence, etc. [36] Kemperman et al. [37] found that loneliness is directly associated with older adults’ social network satisfaction and neighborhood relationships and is indirectly correlated with security perception and community facilities and service satisfaction, which is similar to some of our findings. Liu [18] also discovered that better-quality relationships and emotional intimacy with family and social support peers could facilitate the positive aging of community-dwelling elderly people.

The relationship between MDWs and older adults is an important topic in Hong Kong, alongside the cultural framework of the empty nest period [38]. The average household size in Hong Kong is around two, implying that older adults may be generally living alone with their spouse [9]. Therefore, there is a considerable demand for MDWs in Asian countries, same as in Hong Kong [11,16,17]. According to the cultural framework of the empty nest period, it is important to consider the culturally specific long-term model in Asia countries (e.g., Hong Kong) to support older adults by hiring MDWs. The help of MDWs may further enhance home-based care for older adults by facilitating their social engagement, delaying institutionalized care, or reducing hospitalizations as much as possible [13,20], aligning with our study participants’ expressed wishes. Indeed, most older adults or their families prefer to master their own daily living, preserve independence, and have person-centered care practice at home rather than passively accepting care in institutions or hospitals [13,20].

Our survey interview participants (*n* = 270, 93.8%) reported poor mutuality with their MDWs, evidenced by the MS total score < 2.5. The results also demonstrated that social loneliness negatively and significantly correlated with perceived dyadic relationship quality and social network, but not emotional loneliness. However, perceived dyadic relationship quality is positively and significantly associated with a perceived social network. There were diverse perceived dyad relationships between older adults and MDWs that could have existed [20,21,34], similar to our study findings. For instance, Wang et al. [20] found that the dyad relationship could be varied from as good as family-like trusting relationships to potential neglect or abuse that might happened, which echoed different relationships that emerged from our participants’ narratives, such as family-like companionship bonding or merely employer-employee relations avoid interaction, etc. [20]. Meanwhile, trusting relations and sharing personal experiences between employers and domestic helpers reflect a deep personal connection beyond employment [34]. Indeed, the closeness of relationships could be affected by various reasons, e.g., personal values, cultural beliefs, living arrangements, and employment duration. Moreover, these relationships might have further implications for the health and well-being of older adults, as reflected in our findings.

Besides, research studies illustrated positive experiences among older adults, primary family caregivers, and MDWs [11,13,21,25]. MDWs have developed close or harmonious relationships with older adults and their families [11,25]. They also assisted and fulfilled older individuals’ needs through home-based care [13]. Like our findings, older adults established trusting relationships and shared past experiences and happiness with their MDWs. They also appreciated the home-based personal physical care provided, outdoor social activities they were connected to, and escort services for them to get to healthcare organizations, especially those with mobility incompetence, offered by the MDWs. The role of MDWs and the dyad relationship between older adults and the MDWs could facilitate social activities involvement and receive social support, leading to decreased social isolation and loneliness in older adults.

Though positive experiences and relationships were described, a formal employer-employee relationship or hierarchy might still exist. While maintaining respectful interaction, some employers and employees might prefer clear boundaries with limited personal engagement or discussions [39], similar to some participants in our study with limited personal disclosure and admitting individual roles and privacy. Furthermore, Hoens and Smetcoren [13] emphasize the “authority and control” over the live-in migrant care workers, reflecting a hierarchy. Iecovich [36] found an ambivalent relationship between older Israelis and their primary family caregivers and migrant care workers. The intensity of the ambivalence was significantly related to the loneliness level informed by functionally incapacitated older adults. Our findings also reported participants’ perceived experiences of MDW behaviors negatively impacting the household functionality and the harmony of the dyad quality relationship.

With a rising trend in life expectancy, the older population is expected to increase rapidly to 2.514 million (~30% of the total population) in 2043 [40]. The need for long-term care services in terms of community care services (CCS) and residential care services (RCS) will inevitably increase locally. The home-based community care services provided by local healthcare professionals may not be sufficient for the increasing trend and the challenges of an aging population in recent decades in Hong Kong. The existence of MDWs is paramount, not just in assisting in domestic and personal care at home but also in regularly accompanying the participants to attend follow-up appointments in healthcare organizations. Usually, the MDWs have limited or even no training for long-term care before employment [20]. As MDWs should only perform domestic services locally, employers or care-receiver families should carefully consider whether the work assigned to the MDWs is appropriate or presents risk for both the MDWs and the older adults, especially those physically or psychologically incompetent. The government, non-government organizations, MDW agencies, healthcare organizations, or other related organizations should consider collaborating on appropriate training services and support to make them feasible and lawful and ensure both parties’ safety. With suitable and sufficient training, MDWs may safely provide person-centered care for older adults.

This study collected quantitative and qualitative data at different stages, allowing the researchers to understand both comprehensive and in-depth perspectives of participants’ views. All the surveys and in-depth interviews were conducted face-to-face in quiet rooms to ensure privacy and safety concerns for the participants. Due to the prolonged COVID-19 pandemic, the elderly community centers only provide limited services to older adults, and most activities were organized with prior arrangements to maintain social distancing and avoid cross-infection in the community. In addition, older adults, especially those cared for by MDWs, reduced their visits to the centers. The pandemic largely impeded the feasibility of random sampling of older adults in each center. Therefore, the recruitment method was changed from random sampling to convenient and snowball sampling, and the recruitment period was longer than expected. Besides, those older adults who were referred by the elderly centers and participated in this study might be those who were more active in joining social activities or better social networks, which might lead to selection bias. For the participants’ gender ratio, our female participant ratio (85.8% in survey interviews and 73.7% in in-depth interviews) was far beyond the gender ratio (884 males/1000 females) in Hong Kong [41]. Moreover, our study participants’ MDWs were mainly from Indonesia (survey 78.8% and in-depth interviews 73.6%), which was higher than the data of MDWs from the Hong Kong government (56% Philippines and 42% Indonesia) [19]. It might be related to the employer’s preference for MDWs’ language usage in elderly care, as more Indonesians understand Cantonese [12]. Finally, our findings are culturally specific to the long-term care patterns in Hong Kong. As suggested by Hartanto et al. [38], it is important to be cautious when transferring our findings to other countries with MDWs (e.g., Singapore).

## 5. Conclusions

Our study demonstrated a high prevalence of loneliness among older adults being cared for by MDWs in Hong Kong. Social loneliness negatively and significantly correlated with perceived dyadic relationship quality and social network. The findings also illustrated that good dyadic relationships among MDWs with older adults would enhance older adults’ social connections and networking, relieving loneliness and social isolation. Appropriate strategies to strengthen older adults’ social support and improve the quality of dyadic relationships with MDWs may reduce loneliness among older adults. The government, non-government organizations, MDW agencies, and healthcare organizations are advised to offer extra support and strategies to enhance health and social services for older adults and strengthen in-service or before-employment personal care training of MDWs if appropriate. Further study is suggested to explore the well-being and experiences of older adults and MDWs and the impact of these relationships on their health.

## Figures and Tables

**Table 1:** Socio-demographics, health status, and co-living profile of the participants (*n* = 288).

Characteristics	Mean (SD)/Frequency (%)
Older adults	
Age, years	82.77 (7.72)
Gender	
Male	41 (14.2%)
Female	247 (85.8%)
Marital status	
Single/divorced/separated/widowed	181 (62.9%)
Married	106 (36.8%)
Refused to tell	1 (0.3%)
Educational level	
Nil/pre-primary	67 (23.2%)
Primary	131 (45.5%)
Secondary and above	89 (30.9%)
Refused to tell	1 (0.3%)
Social networks, LSNS	20.09 (8.92)
LSNS < 20	146 (50.7%)
Functional capacity, IADL	12.71 (4.13)
Loneliness, DJGLS score	
Overall score	1.60 (1.67)
Overall score ≥ 2	128 (44.4%)
Emotional subscale score	0.75 (0.96)
Emotional subscale score ≥ 1	131 (45.5%)
Social subscale score	0.85 (1.10)
Social subscale score ≥ 1	129 (44.8%)
Living with family	
No	132 (45.8%)
Yes	156 (54.2%)
Dyadic relationship quality, MS total score	1.47 (0.74)
MS total score < 2.5	270 (93.8%)
Foreign Helper	
Nationality	
Filipinos	53 (18.4%)
Indonesians	227 (78.8%)
Others (including Thais, Malaysians, and Bengalese)	5 (1.7%)
Refused to tell	3 (1.0%)
Spoken language	
Cantonese	234 (81.3%)
Non-Cantonese	53 (18.4%)
Refused to tell	1 (0.3%)
Serving years	3.27 (3.57)

SD—standard deviation.

**Table 2:** Mean, standard deviations, and correlations among the variables.

Variables	Mean	SD	1	2	3	4	5
1. Overall loneliness	1.60	1.67	-				
2. Social loneliness	0.85	1.10	0.83 **	-			
3. Emotional loneliness	0.75	0.96	0.77 **	0.30 **	-		
4. Perceived dyadic relationship quality	1.47	0.74	−0.15 *	−0.18 **	−0.06	-	
5. Perceived social network	20.09	8.92	−0.19 **	−0.20 **	−0.10 *	0.31 **	-

*n* = 288. * *p* < 0.05. ** *p* < 0.01.

**Table 3:** Bootstrapping indirect effects and 95% confidence interval (CI) for the mediation model (overall/social loneliness).

Outcome	Mediator	Effect	Boot SE	95% BC CI	
				Lower	Upper
Overall loneliness	Perceived social network	−0.04	0.02	−0.07	−0.01
Social loneliness	Perceived social network	−0.05	0.02	−0.09	−0.02

**Table 4:** Themes, subthemes, and examples of codes.

Interrelated Themes	Subthemes	Examples of Codes
Established relational interaction with MDWs	Approximate family companionship and care	Resemble family companionship and care Concern about health and well-being Acceptance and interdependence exist
	Trust and sharing initiate emotional attachment	Trustable with good work performance Appreciate loyalty and honesty As a listener and relieve emotions
	Merely employer-employee relations avoid interaction	Fulfil responsibilities or housework Maintain distant relationships or boundaries Avoid unnecessary communication
	Behaviors influence communication and relationships	Unacceptable behaviors Not trustable Lack of communication
Enjoyed functional assistance and support from MDWs	Assisting in daily domestic work	Offer help in daily affairs and housework Relieve workload or burden Dissatisfy with work performance
	Providing personal physical care and support	Support in personal care Offer help to relieve pain Assist in bathing
	Accompanying safety outings for activities	Aid in outdoor activities Accompany to attend healthcare appointments Ensure safety for outings
	(Un)Willing to work outside normal hours	Willing to help at midnight Special arrangement of work during the day off Able to work if required

## Data Availability

The data presented in this study are available upon reasonable request from the corresponding author. They are not publicly available due to privacy or ethical restrictions.
